# Consensus opinion from an international group of experts on measurable residual disease in hairy cell leukemia

**DOI:** 10.1038/s41408-022-00760-z

**Published:** 2022-12-13

**Authors:** Farhad Ravandi, Robert J. Kreitman, Enrico Tiacci, Leslie Andritsos, Versha Banerji, Jacqueline C. Barrientos, Seema A. Bhat, James S. Blachly, Alessandro Broccoli, Timothy Call, Dai Chihara, Claire Dearden, Judit Demeter, Sasha Dietrich, Monica Else, Narendranath Epperla, Brunangelo Falini, Francesco Forconi, Douglas E. Gladstone, Alessandro Gozzetti, Sunil Iyengar, James B. Johnston, Jeffrey Jorgensen, Gunnar Juliusson, Francesco Lauria, Gerard Lozanski, Sameer A. Parikh, Jae H. Park, Aaron Polliack, Graeme Quest, Tadeusz Robak, Kerry A. Rogers, Alan Saven, John F. Seymour, Tamar Tadmor, Martin S. Tallman, Constantine S. Tam, Philip A. Thompson, Xavier Troussard, Clive S. Zent, Thorsten Zenz, Pier Luigi Zinzani, Bernhard Wörmann, Kanti Rai, Michael Grever

**Affiliations:** 1 Department of Leukemia, University of Texas MD Anderson Cancer Center, Houston, TX USA; 2 Laboratory of Molecular Biology, National Cancer Institute, NIH, Bethesda, MD USA; 3 Institute of Hematology, Department of Medicine and Surgery, University and Hospital of Perugia, Perugia, Italy; 4 University of New Mexico Comprehensive Cancer Center, Albuquerque, NM USA; 5 Department of Internal Medicine & Biochemistry and Medical Genetics, Rady Faculty of Health Sciences, University of Manitoba, Winnipeg, MB Canada; 6 CancerCare Manitoba Research Institute, CancerCare Manitoba, Winnipeg, MB Canada; 7 Feinstein Institutes for Medical Research and Zucker School of Medicine at Hofstra/Northwell, New Hyde Park, NY USA; 8 Division of Hematology, Department of Internal Medicine, The Ohio State University, Columbus, OH USA; 9 IRCCS Azienda Ospedaliero-Universitaria di Bologna, Istituto di Ematologia “Seràgnoli”; and Dipartimento di Medicina Specialistica, Diagnostica e Sperimentale Università di Bologna, Bologna, Italy; 10 Division of Hematology, Mayo Clinic, Rochester, MN USA; 11 Department of Lymphoma and Myeloma, University of Texas MD Anderson Cancer Center, Houston, TX USA; 12 The Royal Marsden NHS Foundation Trust, London, UK; 13 Department of Internal Medicine and Oncology, Semmelweis University, Budapest, Hungary; 14 Department of Hematology, University Hospital of Heidelberg, Heidelberg, Germany; 15 Division of Molecular Pathology, The Institute of Cancer Research, London, UK; 16 School of Cancer Sciences, Cancer Research UK Southampton Centre, Faculty of Medicine, University of Southampton, Southampton, UK; 17 Haematology Department, Cancer Care Directorate, University Hospital Southampton NHS Trust, Southampton, UK; 18 Johns Hopkins Kimmel Cancer Center Baltimore, Baltimore, MD USA; 19 Dept. of Medicine, Surgery and Neurosciences, University of Siena, Policlinico S. Maria alle Scotte-, Siena, Italy; 20 Department of Internal Medicine, University of Manitoba, Winnipeg, MB Canada; 21 Department of Hematopathology, University of Texas MD Anderson Cancer Center, Houston, TX USA; 22 Lund University Stem Cell Center, Lund, Sweden; 23 University of Siena, Siena, Italy; 24 Department of Pathology, The Ohio State University Medical Center, Columbus, OH USA; 25 Department of Medicine, Memorial Sloan Kettering Cancer Center, New York, NY USA; 26 Hadassah University Hospital, Jerusalem, Israel; 27 Kingston Health Sciences Centre, Canada, Canada; 28 Department of Hematology, Medical University of Lodz, Lodz, Poland; 29 Division of Hematology and Oncology, Scripps Clinic, La Jolla, CA USA; 30 Haematology Department, Peter MacCallum Cancer Centre & Royal Melbourne Hospital, University of Melbourne, Melbourne, VIC Australia; 31 Hematology Unit, Bnai Zion Medical Center; and the Ruth and Bruce Rappaport Faculty of Medicine, Technion, Haifa, Israel; 32 Department of Haematology, Alfred Hospital and Monash University, Melbourne, Victoria Australia; 33 Department of Hematology, Centre Hospitalier Universitaire Cote de Nacre, Caen, France; 34 James P. Wilmot Cancer Institute, University of Rochester Medical Center, Rochester, NY USA; 35 Dept. of Medical Oncology and Haematology, University Hospital Zürich and University of Zurich (UZH), Zurich, Switzerland; 36 The Charité – Universitätsmedizin Berlin, Berlin, Germany

**Keywords:** Cancer therapeutic resistance, Hairy cell leukaemia

## Abstract

A significant body of literature has been generated related to the detection of measurable residual disease (MRD) at the time of achieving complete remission (CR) in patients with hairy cell leukemia (HCL). However, due to the indolent nature of the disease as well as reports suggesting long-term survival in patients treated with a single course of a nucleoside analog albeit without evidence of cure, the merits of detection of MRD and attempts to eradicate it have been debated. Studies utilizing novel strategies in the relapse setting have demonstrated the utility of achieving CR with undetectable MRD (uMRD) in prolonging the duration of remission. Several assays including immunohistochemical analysis of bone marrow specimens, multi-parameter flow cytometry and molecular assays to detect the mutant *BRAF* V600E gene or the consensus primer for the immunoglobulin heavy chain gene (*IGH*) rearrangement have been utilized with few comparative studies. Here we provide a consensus report on the available data, the potential merits of MRD assessment in the front-line and relapse settings and recommendations on future role of MRD assessment in HCL.

## Introduction

Achieving complete morphological remission has been the first step in achieving cure in cancer therapy. In solid tumor oncology, surgical resection has been typically followed by adjuvant therapy in order to eliminate the circulating and systemic residual tumor cells that are conceptually the cause of relapse and metastatic disease. In hematological cancers detecting measurable residual disease (MRD) has been advocated and has become increasingly important [1]. Such MRD detection is feasible due to the introduction of more sophisticated assays able to identify residual tumor cells in bone marrow and blood specimens [2].

The importance of detecting MRD in various hematological malignancies is dependent on a number of factors that are characterized by the disease biology, availability of effective therapeutic strategies in relapse, the potential need for high risk interventions such as allogeneic stem cell transplantation, as well as by the accuracy and reproducibility of the MRD assay utilized. In acute lymphoblastic leukemia, for example, when the disease is highly fatal and rapidly progressive, and where we now have effective agents able to convert a MRD detectable to undetactable MRD (uMRD) remission in the majority of patients, detection of MRD is increasingly important [3, 4]. In more indolent disorders such as chronic myeloid leukemia and chronic lymphoid leukemia, the recent availability of highly effective therapies, the availability of effective drugs for salvage, and the generally less aggressive nature of the disorders has transformed the role of MRD monitoring from early detection for intervention to a potential indicator of safety of earlier termination of therapy [5, 6].

Hairy cell leukemia (HCL) has been in the forefront of diagnostic and therapeutic advances in leukemia [7]. From early days with its distinct morphology, to more recent description of the almost universal expression of *BRAF* mutations and their contribution to pathogenesis of the disease, HCL has been recognized as a relatively uncommon hematological neoplasm with distinct biological features [8, 9]. Its therapy has also provided the hematologists with a road map of how to incorporate successively more effective treatment options in the standard of care of the patients [10, 11]. These therapeutic advances have resulted in HCL being one of the most manageable hematological neoplasms with long-term disease free survival now becoming a rule and not an exception [12].

MRD detection in HCL is not new, with initial reports of immunohistochemical (IHC) analysis of bone marrow specimens to more recent efforts using multi-parameter flow cytometry (MFC) or polymerase chain reaction (PCR) for detecting the mutant *BRAF* gene [13–15]. In this report, we review the available assays for monitoring disease status in HCL and discuss the potential role of MRD assessment in routine patient care and in clinical trials investigating the relative efficacy of various treatment options in this disease.

### MRD assessment in HCL

As in other hematological cancers, the detection of MRD in HCL has become increasingly relevant because of the development of effective frontline strategies capable of achieving complete morphological remission in the majority of patients [16, 17]. With the introduction of the nucleoside analogs (NA) cladribine and pentostatin, over 75–90% of newly diagnosed patients with HCL can achieve complete remission (CR) [18, 19]. Unfortunately, relapse rates after a single course of NA increase the longer patients are followed, climbing to 47–48% by 15 years of follow-up [20]. Although especially with the availability of new and effective agents, many patients achieve second and subsequent remissions (including after retreatment with a prior regimen), these are generally of a lesser quality and of shorter duration [20, 21]. The median age at diagnosis of patients with HCL is in the 50 s and therefore strategies to improve the quality and duration of first response and potentially cure patients are desirable [22]. Therefore, even in the early studies of NAs, there was significant interest in detecting residual leukemia and predicting likelihood of relapse based on MRD.

Investigators from the Northwestern University Medical School were the first to report that using IHC with B-lineage antibodies L26 and MB2 in fixed bone marrow biopsy specimens, it was possible to detect residual HCL in patients in CR after therapy with cladribine [13]. In a follow-up study, paraffin embedded bone marrow biopsies from 39 patients with HCL, in CR at least three months after a single course of cladribine, were examined by routine hematoxylin and eosin (H&E) staining and IHC using anti-CD45RO, anti-CD20 and DBA44 [23]. Patients with detectable MRD at any time after therapy were more likely to relapse than patients with uMRD (*P* = 0.016) suggesting the potential value of MRD assessment in predicting relapse [23, 24]. In a similar study, Ellison and colleagues used IHC with antibodies to CD20 and DBA44 to evaluate 154 bone marrow specimens obtained between 3 and 25 months after therapy with cladribine for presence of residual hairy cells [25]. They categorized bone marrow findings into negative, indeterminate (IHC stains positive but without morphological features), rare, <1%, 1–3%, 3–5%, and >5% of total cell population. The distinction between rare and indeterminate was on the basis of presence of at least 5 cells with HCL morphology staining positive for CD20 or DBA44 [25]. The proportion of biopsies positive for MRD was similar over the 25 month follow-up period suggesting the stability of amount of residual disease (Table 1), with only 4 of the 18 patients having multiple biopsies showing an increase in percentage of hairy cells over time.

**Table 1:** Immunohistochemical detection of MRD in Bone marrow after cladribine (Adapted from Ellison, DJ, Blood, 1994).

Months post-therapy	Positive *N* (%)	Indeterminant *N* (%)	Negative *N* (%)
3–4	18 (47)	19 (50)	1 (3)
5–7	17 (51)	11 (39)	0
8–10	9 (53)	7 (41)	1 (6)
11–13	20 (43)	25 (53)	2 (4)
14–16	5 (50)	5 (50)	0
17–25	10 (56)	6 (33)	2 (11)

Other investigators have utilized IHC for the detection of residual HCL. The investigators from the Swiss group for clinical cancer research examined bone marrow specimens collected at 3, 6, 9, and 12 months after one cycle of subcutaneous cladribine in 17 patients with HCL who had at least 12 months follow-up [26]. Using IHC for DBA44 and CD20, they defined three patterns of MRD ranging from rare scattered suspicious hairy cells at less than 1%, to MRD levels between 1% and 5%, with a third group having MRD levels greater than 5% and suggested that such quantitation of residual hairy cells could help predict the risk for relapse [26]. The recent availability of an antibody specific to mutant *BRAF*-V600E protein, can potentially improve IHC detection of hairy cells in the MRD setting, as in contrast to other IHC markers, this antibody does not stain normal mature B-cells [27]. However, the feasibility of direct molecular detection of mutant *BRAF*, as discussed later, may limit future interest in such assays.

MFC has been more recently employed to detect residual HCL in the bone marrow and peripheral blood and may have an advantage over IHC due to significantly higher number of cells analyzed. Matutes and colleagues used MFC in a cohort of 23 patients with HCL treated with pentostatin to examine peripheral blood and bone marrow samples collected at a median of 10 months after therapy and reported an overall incidence of 43% of detectable MRD [28]. They were unable to show a correlation between persistence of MRD and likelihood of relapse, but the median follow-up was only 72 months.

Investigators at the University of Texas, MD Anderson Cancer Center used 8 weekly doses of rituximab after a cycle of cladribine in 13 patients with newly diagnosed (*n* = 11) or first relapsed HCL (*n* = 2) [15]. All patients achieved a morphological CR and MRD assessed by MFC was negative in 12 of 13 patients after the completion of all therapy (3 months). In a follow-up study, Chihara et al reported the long-term outcome of 59 newly diagnosed patients with HCL, 7 patients with variant HCL, and 14 patients treated at first relapse [29]. Overall, after completion of rituximab therapy, 100% of patients with classical HCL (untreated or relapsed) achieved morphological CR, with 76% (untreated) and 64% (relapsed patients) respectively achieving uMRD in bone marrow aspirate specimens. Moreover, 16 patients had follow-up MRD assessment by MFC in peripheral blood and became undetectable; therefore 75 (94%) achieved an uMRD state. The median time to an uMRD state (bone marrow and/or peripheral blood) was 2.9 months (range, 0.8–18.9 months) [29]. MRD status at any time point was not associated with EFS as there were very few relapses in the total population. However, the few patients without confirmed uMRD status did not relapse.

In phase 2 randomized trial, investigators from the National Institute of Health randomly assigned 68 patients with purine-analog naïve classical HCL to receive cladribine either with rituximab given concurrently (CDAR) or ≥6 months later after detection of MRD in peripheral blood (delayed rituximab) [17]. At 6 months after initiation of cladribine, the CR rate was 88% in the delayed rituximab arm vs. 100% in the concurrent (CDAR) arm (*p* = 0.11). MRD was assessed by MFC. The bone marrow uMRD CR rates were 24% vs 97% (*p* < 0.0001) and peripheral blood MRD clearance was achieved in 50% vs. 100% (p < 0.0001). Since rituximab could not be given until MRD was detected in blood, and blood was never positive for MRD before bone marrow, the durability of MRD-free CR could be determined in these 2 groups with bone marrow performed yearly for 2.5 years after cladribine and biannually thereafter. Durability of MRD-free CR was higher after CDAR than after CDA, with 3% vs 64% of uMRD patients having MRD recurrence during 6.5 years of follow-up (*p* < 0.0001). Delayed rituximab, administered to patients with detectable MRD in blood at least 6 months after cladribine, was able to achieve CR with uMRD in 14 (67%) of 21 patients, and although the durability of CR with uMRD was inferior after delayed rituximab than after CDAR (*p* = 0.0081), most (71%) of the 14 patients remained with uMRD after delayed rituximab during the 6.5 year median follow-up time [17]. Aside from achieving uMRD, an important goal of the study was to prevent or delay relapse requiring retreatment. Of the 68 patients randomized, only 1 (1.5%) patient relapsed with cytopenias during the median 6.5-year follow-up time, compared to 28% of 90 historical patients relapsing with cytopenias at 6.5 years (*p* < 0.0001) [20]. Thus, whether administered in concurrent or delayed fashion, cladribine and rituximab may not only achieve CR with uMRD, but may also prevent or delay relapse of cytopenias requiring treatment.

While the clinical benefit of CR with uMRD requires long-term follow-up to demonstrate in first line treatment of classical HCL, the situation is markedly different in variant HCL (HCLv), where patients have a reduced overall survival (OS) (4–6 years) and are less responsive to purine analog monotherapy, with CR reported in only 8% of 42 reported cases. In a recently published study, 20 patients with HCLv (8 treatment naïve), received concurrent CDAR with 95% achieving CR and 80% achieving CR with uMRD [30]. There was a strong PFS and OS advantage to achieving uMRD by MFC in the bone marrow by 4 weeks (*p* = 0.022–0.025) and by 6 months (*p* < 0.0001) after treatment. PFS and OS advantages were also observed with uMRD by MFC of blood by 4 weeks (*p* = 0.0031–0.0017) and 6 months (*p* < 0.0001). Thus, with HCLv which is more aggressive and less responsive than classical HCL, achievement of CR with uMRD is critical.

**Fig. 1:**
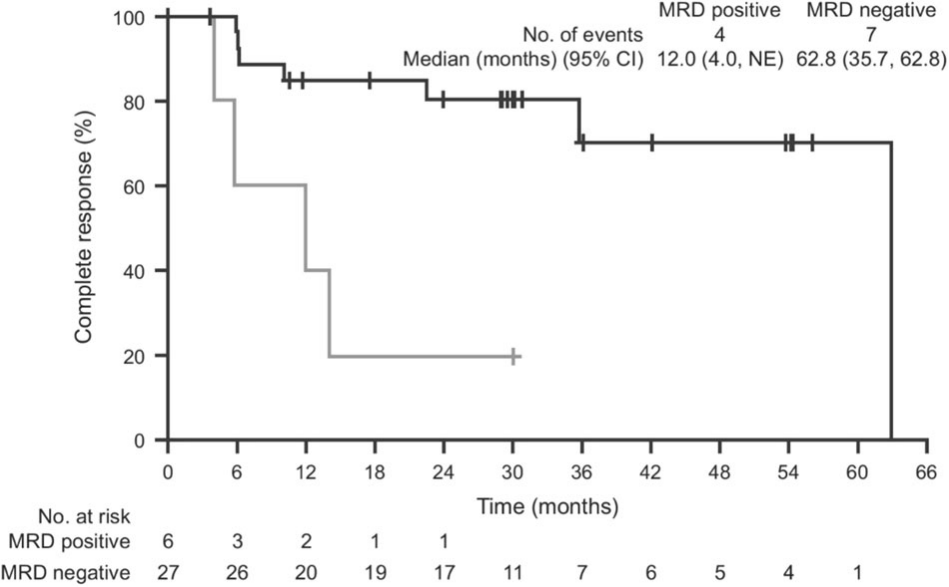
Achievement of MRD negative status by MFC after moxetumomab was associated with prolonged CR duration (Adapted from Kreitman R, et al, J Hematol Oncol, 2021). MRD negativity was associated with durable CR.

Another study supporting the potential utility of MRD assessment by MFC in predicting the risk of a future relapse in patients with relapsed classical HCL was conducted by Kreitman and colleagues. 33 patients with relapsed/refractory HCL were treated with moxetumomab pasudotox (an anti-CD22 immunotoxin) at a fixed dose level on a phase 1 trial and 21 (64%) achieved CR [31]. Among the 32 patients evaluable for MRD assessment by MFC in bone marrow specimens, the median CR duration was significantly longer in the 11 patients achieving uMRD [42.1 months, range 24.0–69.2] compared to the 9 patients with MRD-persistent CR [13.5 months, range 4.9–42.4] (*p* < 0.0001) [31]. This agent was FDA-approved for relapsed/refractory HCL on the basis of a pivotal phase 3 trial in 80 patients [32]. Long-term follow-up of this trial was reported recently [33]. At a median follow-up of 24.6 months, 27 of 33 patients achieving CR were negative for MRD assessed by bone marrow biopsy IHC. The median duration of CR was longer in patients who achieved uMRD status (62.8 vs. 12.0 months) (Fig. 1) [33].

Molecular assays have also been utilized to detect MRD in HCL. Some investigators have employed a polymerase chain reaction (PCR) assay with capillary electrophoresis, using consensus V primers derived from the framework 1 (FR1), FR2 and FR3 regions of immunoglobulin heavy chain (*IGH*) in combination with either a consensus JH or CH primer to detect MRD in HCL [15]. In another study, Cervetti et al, used PCR with consensus primers for the V-D-J regions of the immunoglobulin heavy chain gene (*IGH*) to detect MRD after therapy with cladribine and evaluate the efficacy of rituximab in eradicating the MRD [34]. Eight of 10 patients (2 in CR, 4 in partial remission and 2 unresponsive to cladribine) were evaluable for response and all achieved CR after completion of rituximab. PCR analysis was conducted at 2, 6, and 12 months after the end of rituximab and showed a progressive increase in the proportion of patients in molecular remission to 100% at 1 year suggesting the efficacy of rituximab in eradicating the MRD [34]. This assay may not be sufficiently sensitive and has not been extensively utilized. However, using patient specific primers and a patient specific probe with reporter and quencher, it was possible to detect 1 HCL cell in 10^6^ normal cells [35], and observe MRD in patients who had uMRD by MFC, similar to highly sensitive MRD detection in patients with acute lymphoblastic leukemia [36]. While this method requires sequencing the immunoglobulin heavy chain rearrangement prior to treatment, it is also possible to use PCR to detect genes which are highly sensitive for HCL, like *MYF6* [37]. A TaqMan PCR assay using primers and probe for MYF6 could detect 10 HCL cells in 10^6^ normal cells.

Sausville and colleagues evaluated 86 peripheral blood specimens from 24 patients with HCL [38]. Paired analysis using MFC and consensus primer PCR for *IGH* gene rearrangements was conducted after treatment for detection of MRD. Monoclonal B-cell populations were detected by PCR in 22 of 86 (26%) whereas MFC detected residual leukemia in 48 of 86 (56%) of specimens. In 21 specimens, both methods were positive whereas in 37 specimens both were negative. MFC detected HCL in 27 specimens negative by PCR and only in 1 specimen, PCR was positive and MFC negative [38]. Similarly, Chihara and colleagues did not find any patients where *IGH* consensus PCR was more sensitive than MFC [17]. However, similar data using clone-specific PCR are not available.

**Fig. 2:**
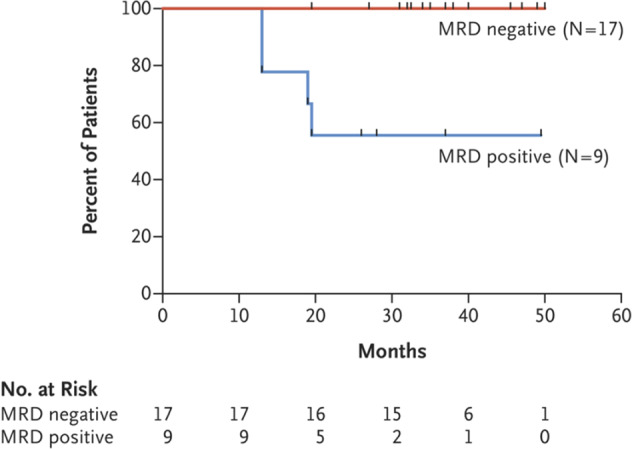
Relapse-free survival by MRD status among patients treated with vemurafenib plus rituximab. (Adapted from Tiacci E, et al, NEJM, 2021).

The detection of a heterozygous mutation in *BRAF* gene resulting in a V600E variant protein in all 48 patients with classical HCL and none of 195 patients with other B-lymphoid leukemia or lymphomas was a remarkable cornerstone in defining the pathogenesis of this leukemia and led to trials investigating the role of BRAF inhibitors in HCL [9, 11, 39] Initial trials investigated the potential role of monotherapy with the BRAF inhibitor, vemurafenib and demonstrated the significant efficacy of the small molecule inhibitor in treating patients with relapsed HCL [11]. More recently, Tiacci and colleagues reported on a phase 2 trial combining vemurafenib with rituximab in patients with relapsed HCL (median of 3 prior therapies) and reported complete morphological response in 26 of 30 patients enrolled (87%) [14]. They utilized an allele-specific PCR for *BRAF* V600E mutants with a sensitivity of ≥0.05% mutant copies to detect MRD in bone marrow and peripheral blood samples [40]. Responses including evaluation for MRD were performed after two cycles of vemurafenib plus rituximab and at the completion of all therapy including additional 4 doses of rituximab. MRD by PCR for *BRAF* V600E was undetectable in the bone marrow and peripheral blood of 17 of 26 (65%) patients with CR and 18 of 30 patients overall [14]. Nine patients had uMRD after the initial 2 cycles of combined therapy and in an additional 7 patients uMRD by PCR was achieved after the subsequent rituximab therapy. Two patients had persistent MRD at the end of treatment but became negative subsequently with further follow-up. Patients who were in CR after one cycle of the combined therapy were more likely to achieve MRD clearance at the end of treatment [13 of 15 patients (87%) compared to 2 of 9 (22%)] [14]. Among the patients who achieved CR, all of the 17 patients who achieved uMRD by PCR as compared to 56% of 9 patients who had detectable MRD, remained without cytopenia relapse at a median follow up of 34 months (range, 13–50)(Fig. 2) [14]. Survival without MRD recurrence was 100% at a median follow-up of 28.5 months (range, 21–50) among the 17 patients who achieved a uMRD CR. These data suggest that achieving an uMRD status by PCR for *BRAF* V600E may correlate well with freedom from relapse in patients with previously treated HCL.

Two recent studies have utilized digital droplet PCR (ddPCR) for the detection of *BRAF* V600E mutation in HCL [41, 42]. Guerrini and colleagues used the assay in 27 patients with HCL as well as 2 with HCLv and 18 with splenic marginal zone lymphoma and concluded that the assay was more sensitive than quantitative PCR and as specific and therefore very useful for detecting MRD [41]. Similarly, Broccoli and colleagues measured the burden of *BRAF* V600E mutant in peripheral blood or bone marrow specimens from 35 patients with HCL at various stages of disease and reported mean values of fractional disease burden at diagnosis, relapse and response to be 12.26%, 16.52%, and 0.02% in peripheral blood and 23.51%, 13.96%, and 0.26% in bone marrow. The mean value in peripheral blood among 14 patients with long-standing CR was 0.05% including 10 patients who were negative by the assay [42]. These results suggest that ddPCR for *BRAF* V600E may be a useful assay for long-term monitoring of the disease. However, it should be noted that patients with HCLv and a minority of patients with classic HCL lack the *BRAF* V600E mutation [43, 44].

### Pros and cons of available techniques for MRD analysis

Currently, techniques most amenable for MRD detection in HCL are MFC, allele-specific PCR for mutant *BRAF* and IHC.(Table 2) Targeted next-generation sequencing may also be considered, keeping in mind that it cannot reliably detect variant allele frequencies below 1% unless complex and costly strategies such as in silico error correction or molecular barcoding are implemented. These assays have different strengths. MFC and quantitative or digital PCR are significantly more sensitive than IHC, which typically relies on manually counting a few hundred total cells versus hundreds of thousands of events analyzed by MFC or PCR. The sensitivity of the later assays is limited mainly by the input material and can reach 10^–6^ range. However, MFC and PCR require high-quality first-pull marrow aspirate samples, as any hemodilution can jeopardize correct quantification of MRD in the marrow, even to a greater extent than in other leukemias, partly due to the limited peripheral blood involvement by HCL. Short-amplicon PCR applied to formalin-fixed decalcified biopsies may potentially overcome these issues but is yet to be evaluated in the context of HCL MRD. Ultimately, any of the above assays can be utilized to detect MRD in HCL as long as prospective validation with a specified threshold at a specific time-point can demonstrate their prognostic value, or their ability to guide therapy.

**Table 2:** Selected studies/assays utilized for MRD assessment in HCL.

Assay	Study reference	Patient *N*	Disease status	Disease status	Summary
IHC	Tallman et al. [24]	66	Frontline	Cladribine or pentostatin	4-year relapse-free survival superior in uMRD (88% vs. 55%; *p* = 0.0023
MFC	Chihara et al. [29]	59 14	Frontline and Relapsed	Cladribine followed by rituximab	No association of MRD with risk of relapse as few relapses
MFC	Chihara et al. [17]	68	Frontline	concomitant or delayed rituximab (after 6 months)	At 6 months uMRD higher in concomitant (97% vs. 24%; *p* < 0.0001) At 96 months median follow-up lower rate and durability of uMRD CR for delayed rituximab
MFC	Kreitman et al. [31]	33	Relapsed	Moxetumomab Pasudotox	Longer CR duration in patients with uMRD (Median 42.1 months vs. 13.5 months; *p* < 0.001)
MFC	Kreitman et al. [33]	80	Relapsed	Moxetumomab Paseudotox	Longer CR duration (62.8 vs. 12.0 months) in patients achieving uMRD
PCR for mutant BRAF	Tiacci et al. [14]	30	Relapsed	Vemurafenib plus rituximab	Higher relapse-free survival in patients with uMRD CR (100% VS. 56% at median 34 months follow-up
PCR for consensus *IGH* primer(cpPCR)	Sausville et al. [38]	24	Relapsed	various	MFC is superior to cpPCR for detecting MRD in HCL
PCR for clone-specific *IGH* primer (csPCR)	Aarons et al. [35]	10	Relapsed	BL22	csPCR was more sensitive than cpPCR and MFC for detecting MRD
ddPCR for mutant BRAF	Broccoli et al. [42]	36	Frontline and Relapsed	Cladribine	ddPCR is a sensitive assay for quantitation of disease burden and detection of MRD in morphological CR

### Timing of MRD analysis

The timing of the assessment of MRD continues to be subject for debate in various hematological cancers. However, there is general consensus on when MRD assessment is most likely to be predictive of outcome in diseases where the practice is more established such as in acute leukemias, typically at the time of achievement of CR and at the end of consolidation. In HCL, there is very limited available data, and in the studies conducted to date, there is significant variation on the time when a repeat bone marrow exam is performed to establish CR and hence to evaluate the presence or absence of MRD. This ranges from one month after the initiation of therapy with a nucleoside analog, to 3–6 months or even longer. [15, 17–19, 45].

Furthermore, Sigal and colleagues identified 19 patients among the 358-patient Scripps clinic cladribine database who had remained in continuous CR at a median time of 18 years (range, 12–28 years) from diagnosis and 16 years (range, 11–21 years) from cladribine therapy and performed a bone marrow aspiration and biopsy; in nine of 19 (47%) specimens did not show MRD assessed by immunostaining, MFC and/or *IGH* PCR [46]. Morphological evidence of residual HCL was seen in 3 of 19 (16%) patients and MRD was detectable in 7 of 19 (37%) of patients. All patients had normal peripheral blood counts with no other clinical manifestations of HCL [46]. This study suggests that patients with persistent MRD or even morphologically detectable disease are able to survive without clinical relapse for many years after a single course of cladribine monotherapy. Since these patients had not been followed regularly during the median 16-year follow-up time since cladribine, it is possible that some patients had uMRD shortly after treatment but had recurrence of MRD shortly before becoming morphologically positive by bone marrow. Nevertheless, this study raises the question of the utility of MRD assessment in patients with newly diagnosed HCL in determining their long-term relapse-free survival.

On the other hand, relapse in HCL is associated with inferior quality and duration of a second response to therapy with a nucleoside analog, cladribine or pentostatin [20, 21]. Despite the availability of newer effective regimens for treating relapse, improving the quality of the first CR has been debated and MRD assessment can potentially help in this regards [17]. Similarly, in the setting of relapsed disease, MRD status may be an excellent indicator of the efficacy of the salvage regimen and provide very useful information regarding the long-term efficacy of the therapeutic modality [11, 31]. The timing of the MRD assessment in the relapse setting has not been established either. However, it can be argued that MRD monitoring in this setting is more relevant as the CR duration using established regimens is generally limited and any novel strategy capable of producing deeper responses is likely to be desirable.

### Recommendations for MRD monitoring in routine practice and in clinical trials

Based on the available data, assessment of MRD status at the time of achieving response in patients with HCL can be an indicator of the depth of response and potentially a predictor of the duration of remission. It can be debated that in an indolent disorder and with the availability of highly effective initial therapy whether MRD monitoring is essential and whether it can lead to unnecessary patient anxiety and be associated with excessive procedures such as repeat bone marrow evaluations to detect/monitor MRD. Furthermore, although it has been established that responses less than CR are associated with shorter disease-free survival [12], there are no randomized data demonstrating that achieving an uMRD status in first-line affects relapse rates. However, the randomized phase 2 trial in the first line setting comparing concurrent cladribine plus rituximab to delayed rituximab showed that either approach was associated with low risk of relapse cytopenias requiring retreatment (0–3%) [17] compared historically to approximately 30–40% relapsing with cytopenias after purine analog monotherapy during similar follow-up time [47]. Large, randomized trials comparing different therapeutic strategies such as combinations of cladribine or vemurafenib with rituximab with appropriate MRD assessment by MFC and by PCR or IHC for *BRAF* V600E mutation may provide the necessary data for an emphatic stance on the role of MRD monitoring in initial therapy of HCL in the future but may be unrealistic.

However, in the relapse setting, as there are significant long-term data suggesting inadequacy of a second course of a nucleoside analog alone, assessment of the depth of response can be used as a surrogate to compare the relative efficacy of various salvage strategies including chemoimmunotherapy, BRAF inhibitor combinations or immunotoxins. A number of studies in relapsed HCL have already demonstrated the benefit of an uMRD CR for improving the duration of response, further suggesting the utility of this approach [14, 31]. Therefore, we suggest that all trials in the relapse setting should incorporate MRD monitoring, using both MFC and PCR or IHC for *BRAF* V600E as a component of response assessment.

Clearly, as is the case in other hematological cancers, harmonization and standardization of MRD assays among the participating centers is an important step towards further establishing MRD assessment as a routine practice in managing patients with HCL. The rationale for the concurrent administration of cladribine and rituximab is based upon the reported marked reduction in MRD compared to delayed administration of rituximab. In a recent report of this randomized trial of concurrent cladribine and rituximab versus delayed rituximab in 68 patients, the only toxicity which was different in the 2 groups was reversible thrombocytopenia in the concurrent arm, not associated with significant bleeding. (However, 1/3 of the patients required platelet transfusion for platelet count in 8000 to 10,000 range) Neutrophil and platelet recovery were rapid in either arm although at 4 weeks, the concurrent arm was superior with respect to both neutrophils (*p* = 0.017) and platelets (*p* = 0.0015). Safety and efficacy of concurrent CDAR as initial therapy is being further studied with continued follow-up and in additional patients. The 2021 NCCN guidelines now list rituximab as an option with cladribine for 1st-line therapy, either concurrent or delayed. The potential impact of concurrent or delayed rituximab on the humoral immune system may also be highly relevant in this era of widespread infection with SARS CoV-2. The timing of the combined therapy of these agents may impair effective vaccination strategies, so patients should be vaccinated with demonstrable antibodies (if feasible) before starting rituximab as part of any regimen. If, prior to CD20 antibody therapy, patients cannot achieve optimal spike antibody levels with vaccination due to HCL-related deficiency in normal B-cells, one can consider treatment with a BRAF inhibitor to improve counts and normal B-cells, vaccinate, and reconsider CD20 antibody later, once treatment is again indicated and spike antibodies are optimally high (recognizing that testing for such antibodies is not widely available and there are no definitive data on what clinically protective antibody levels may be).

The rare nature of this chronic leukemia requires future cooperative studies engaging multiple institutions to establish the value of MRD testing in the front-line setting. This evaluation of MRD could be incorporated into a randomized trial that would also evaluate the benefit and toxicities of combination therapy in the front-line setting either with cladribine and rituximab or newer approaches involving targeted agents (e.g., a BRAF inhibitor) with rituximab. While enormous progress has been made in the management of classic hairy cell leukemia following the introduction of purine analogs, the time has arrived to evaluate the value of determinations of MRD as a measure of the quality of response to enhance the selection of therapeutic combinations of agents in the treatment of this disease.

## Data Availability

All data generated or analysed during this study are included in this published article and its supplementary information files.
